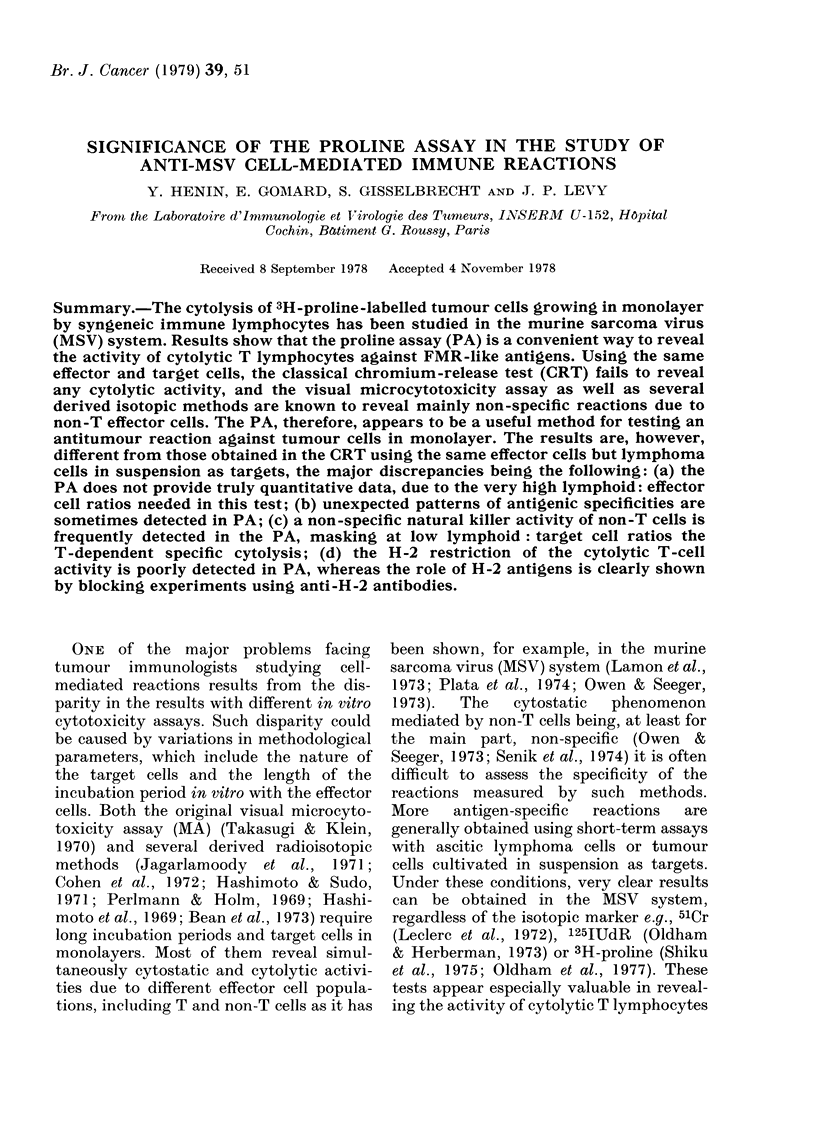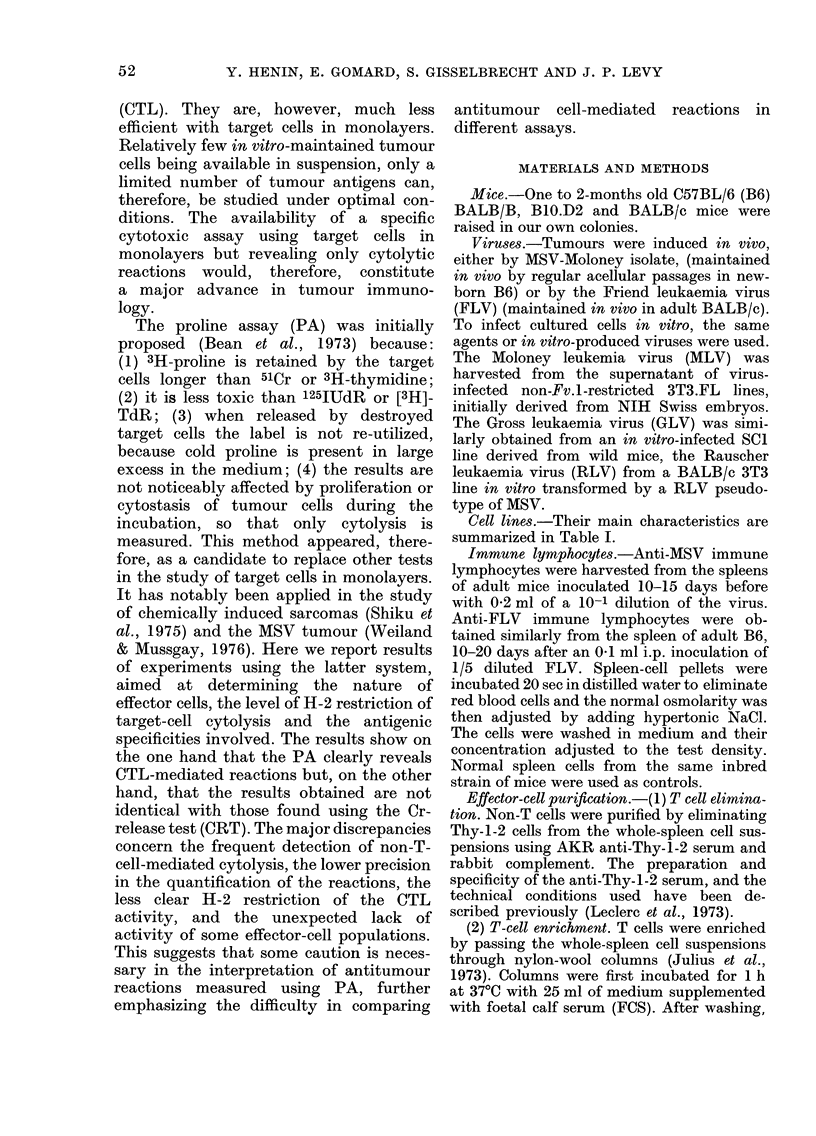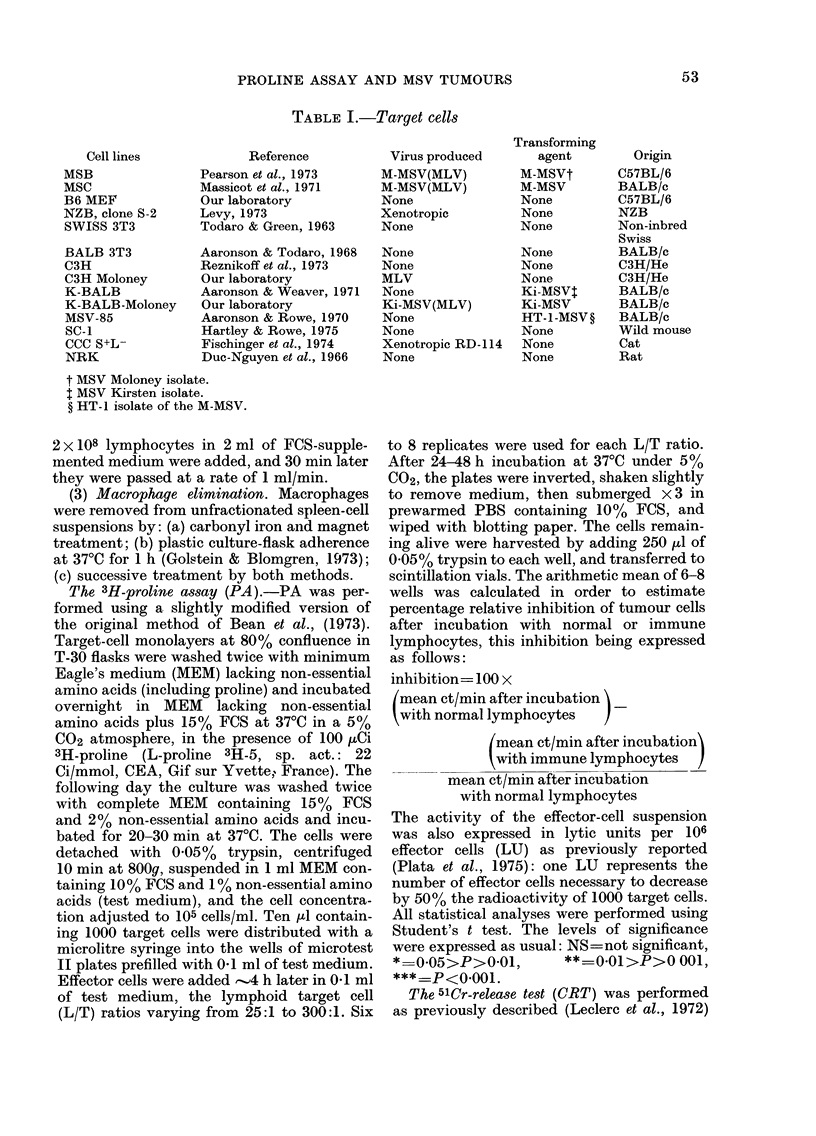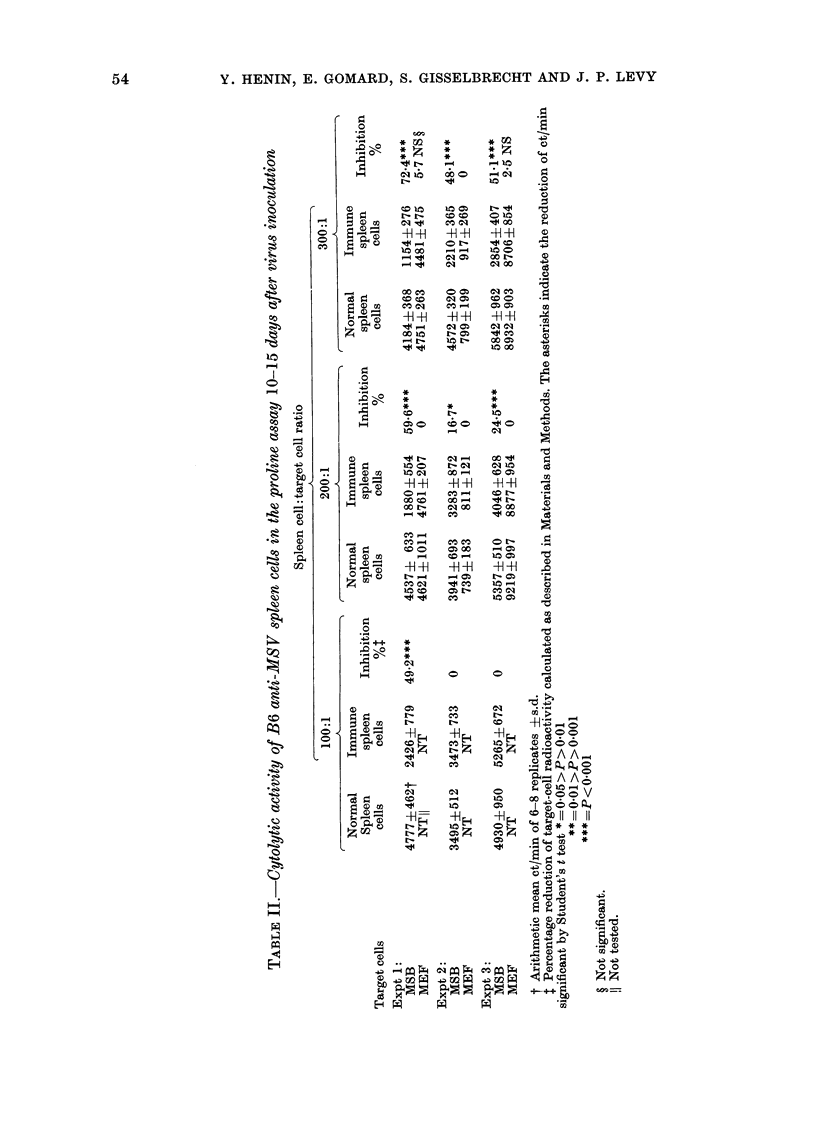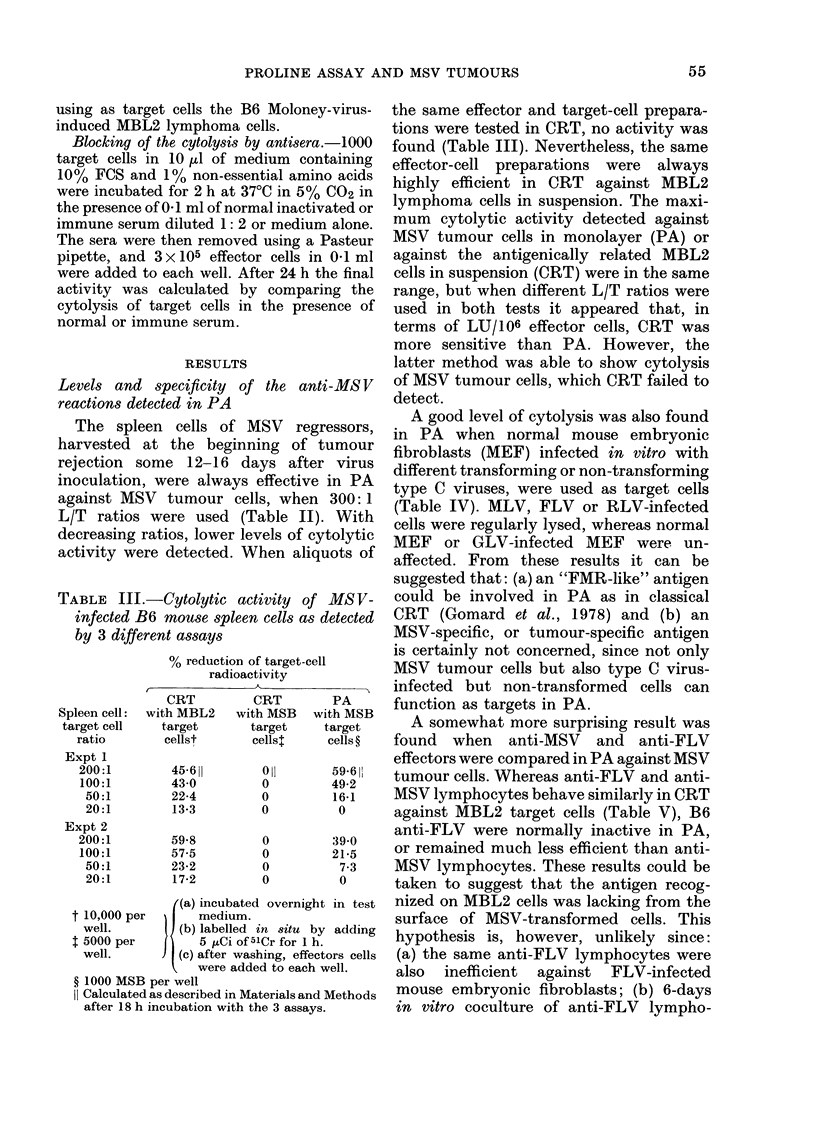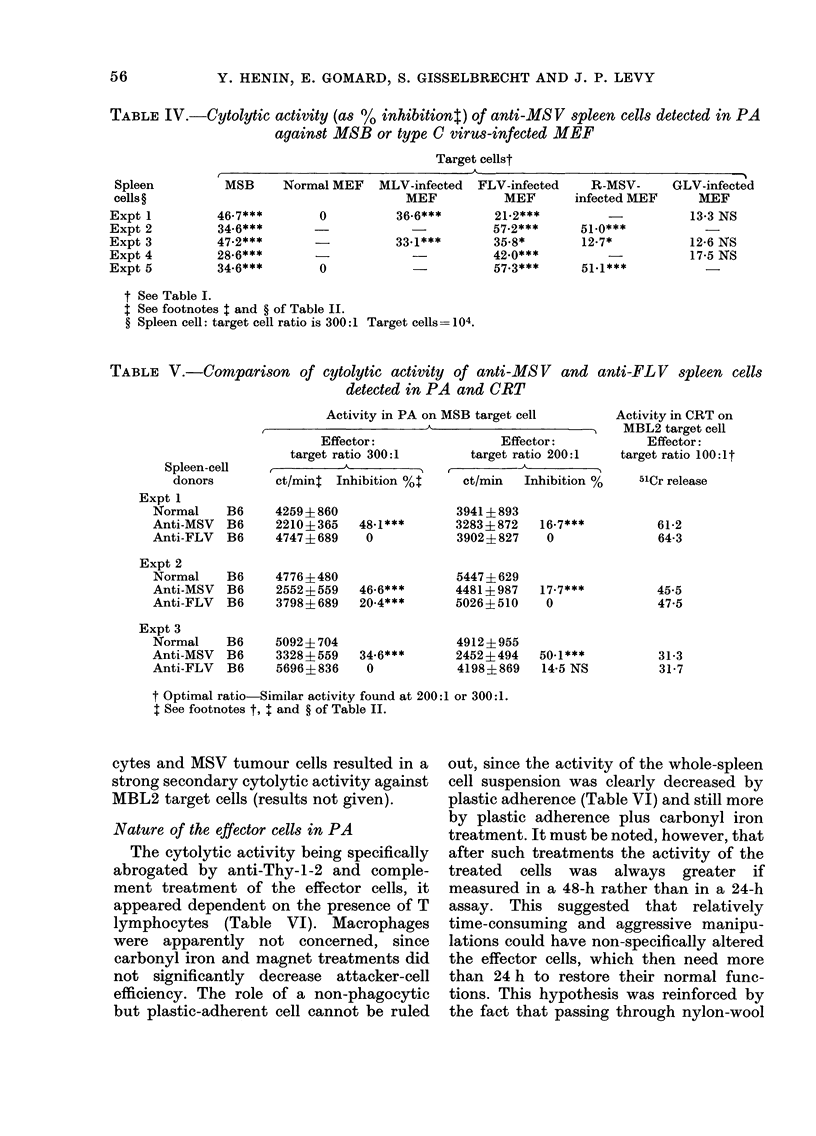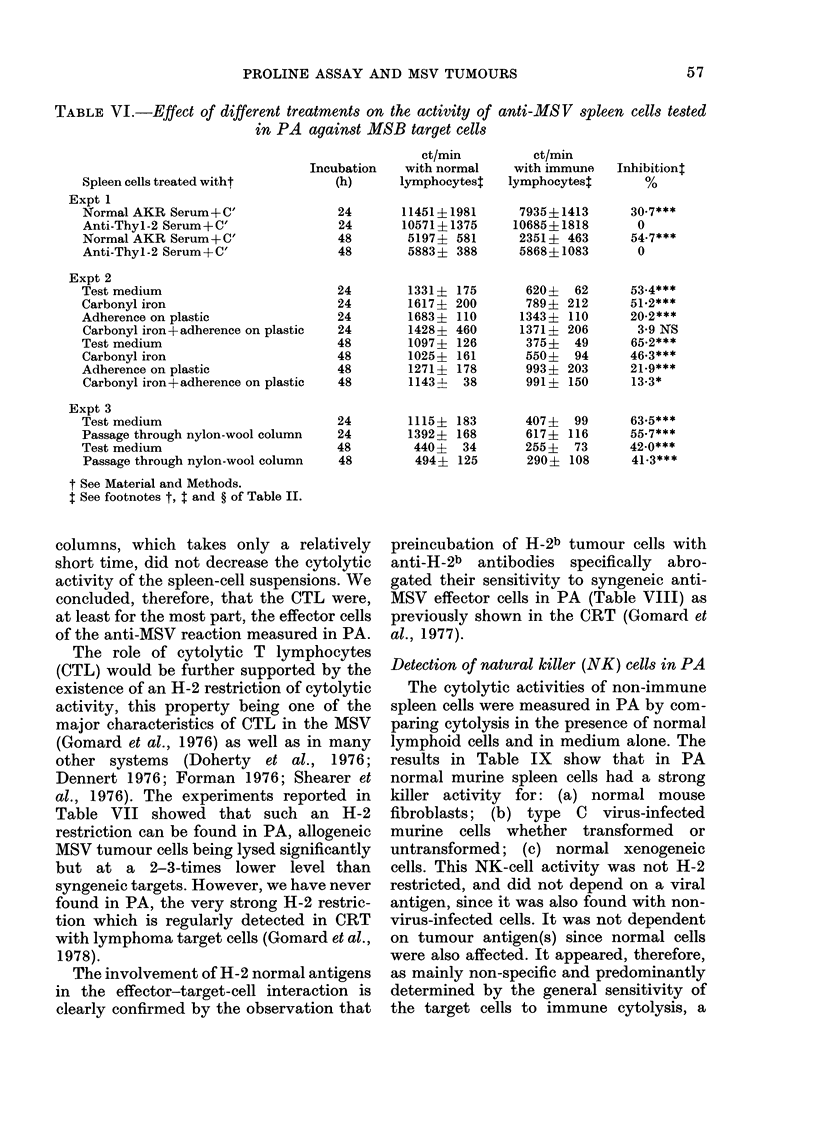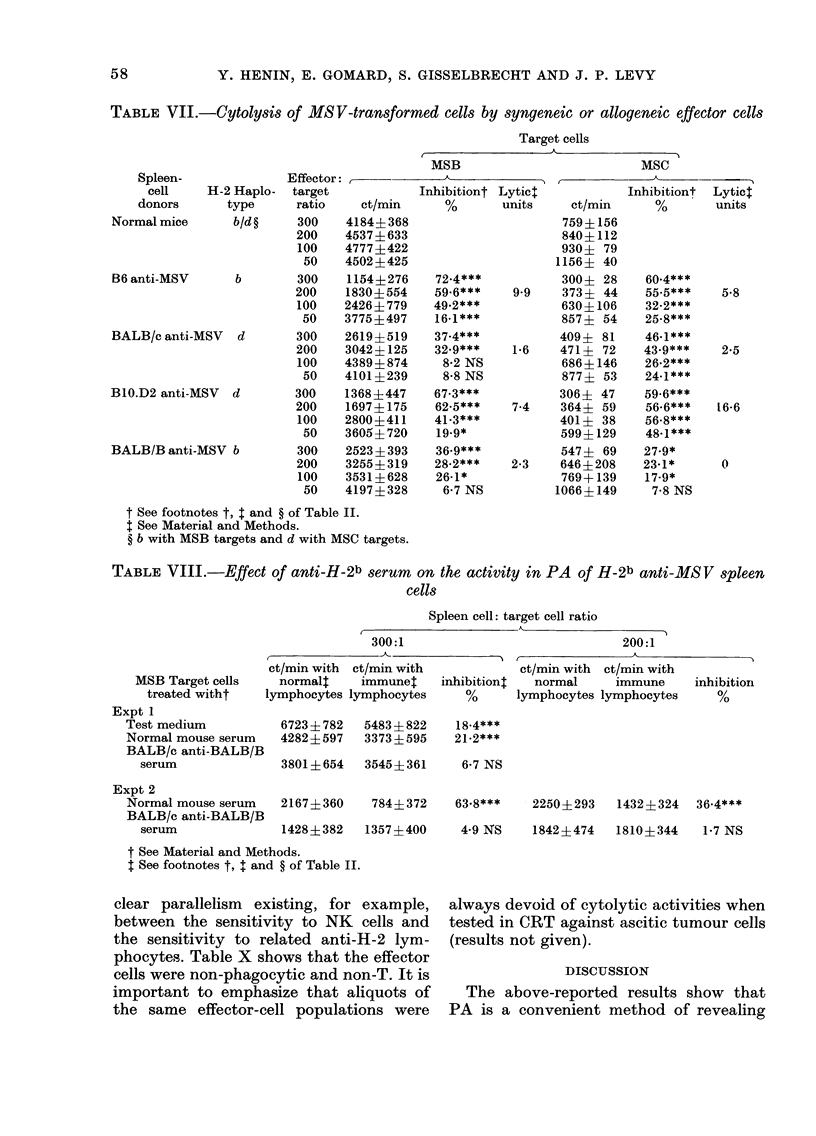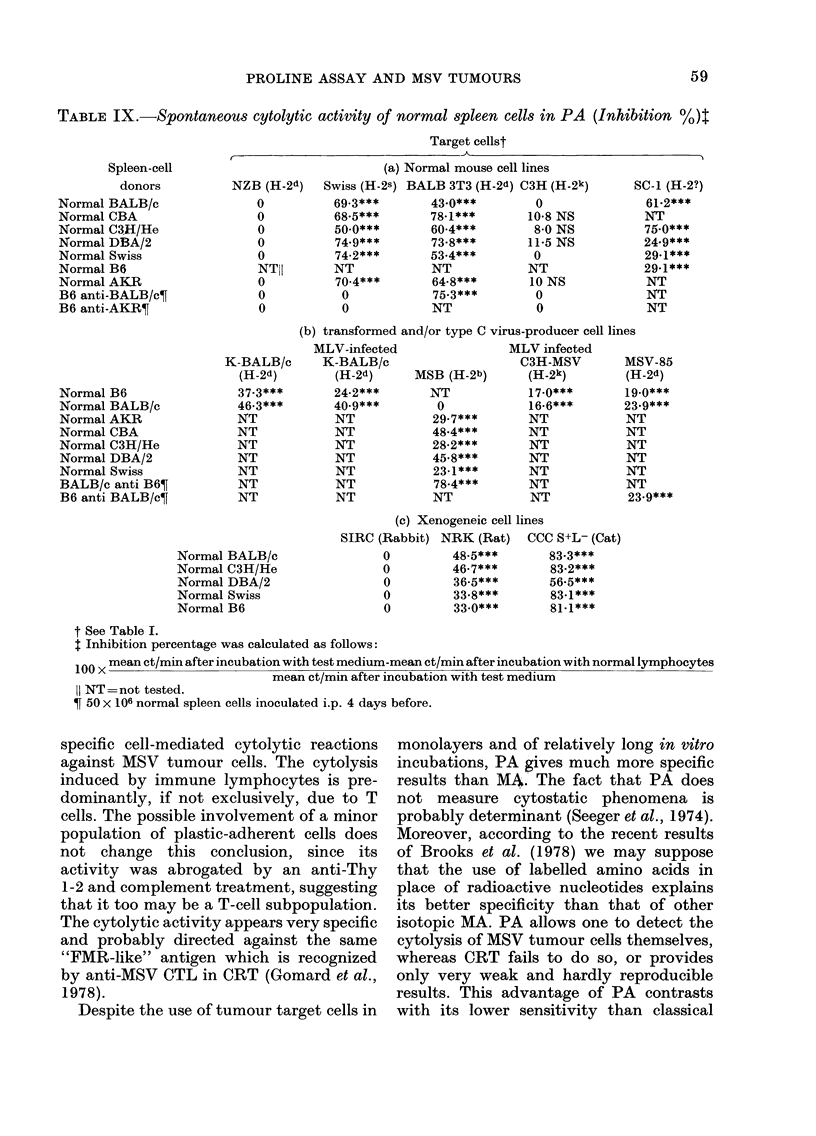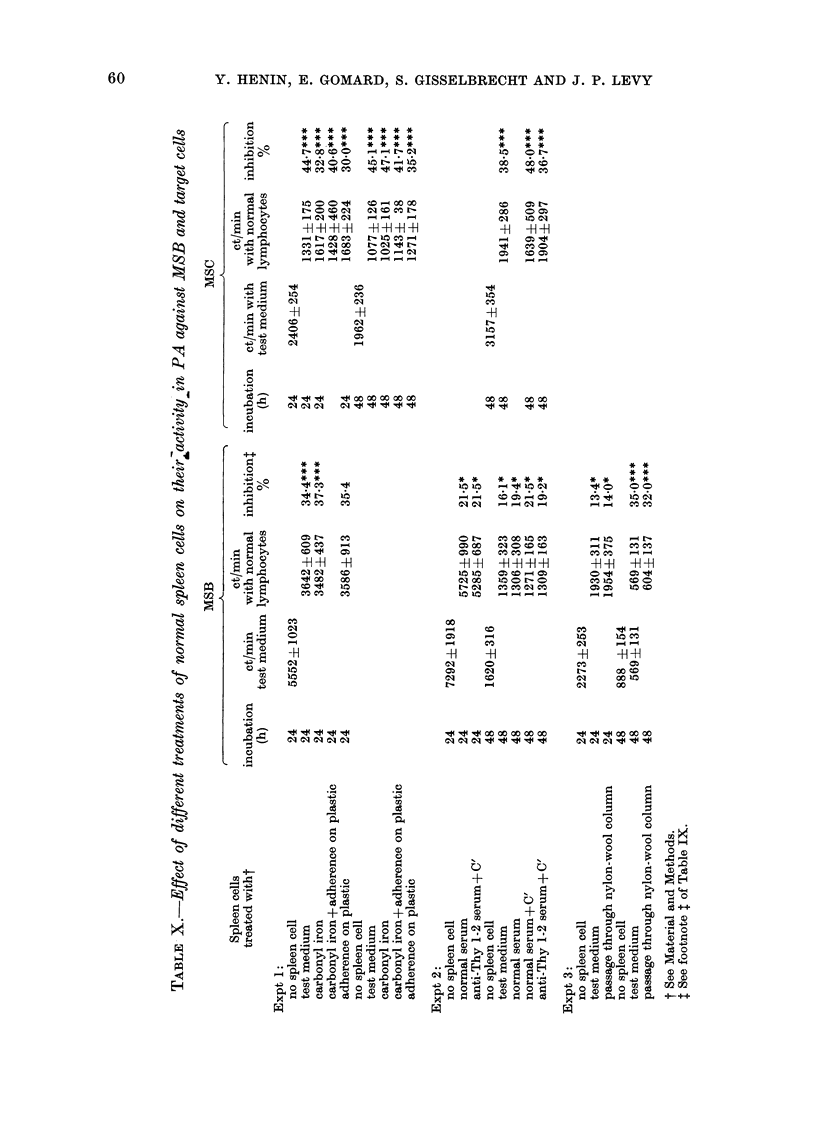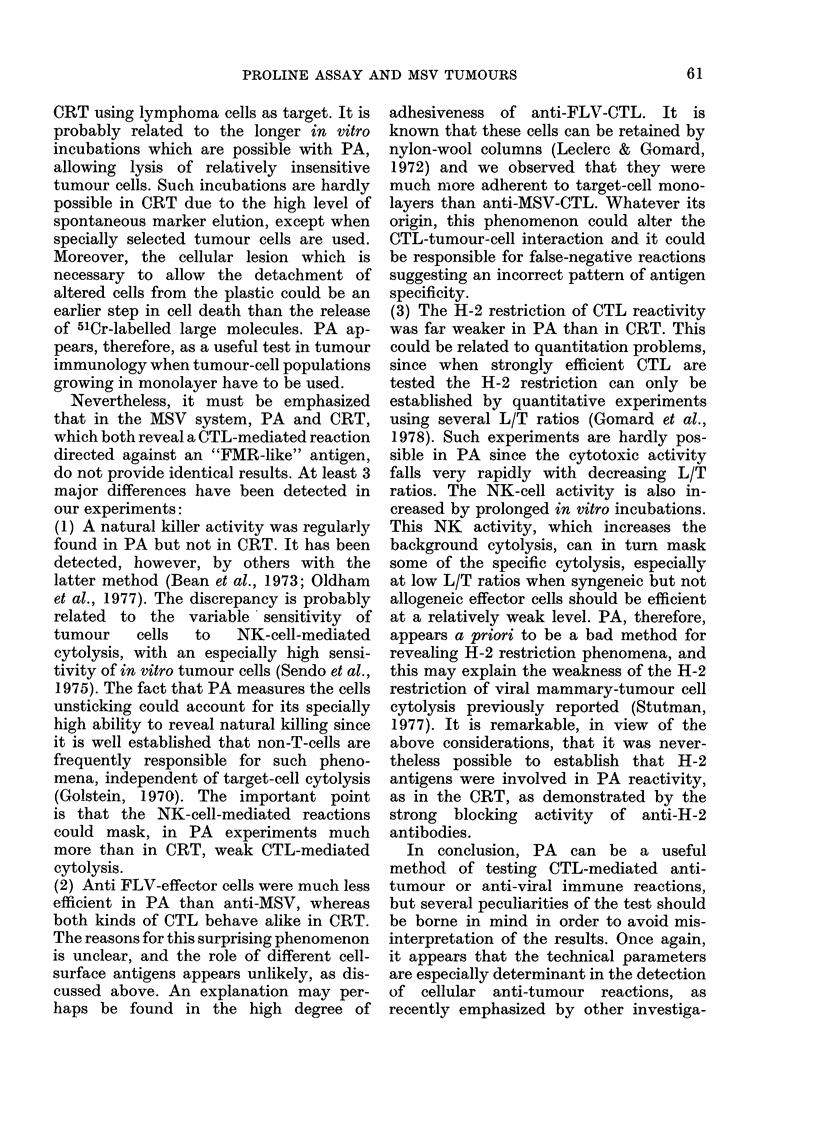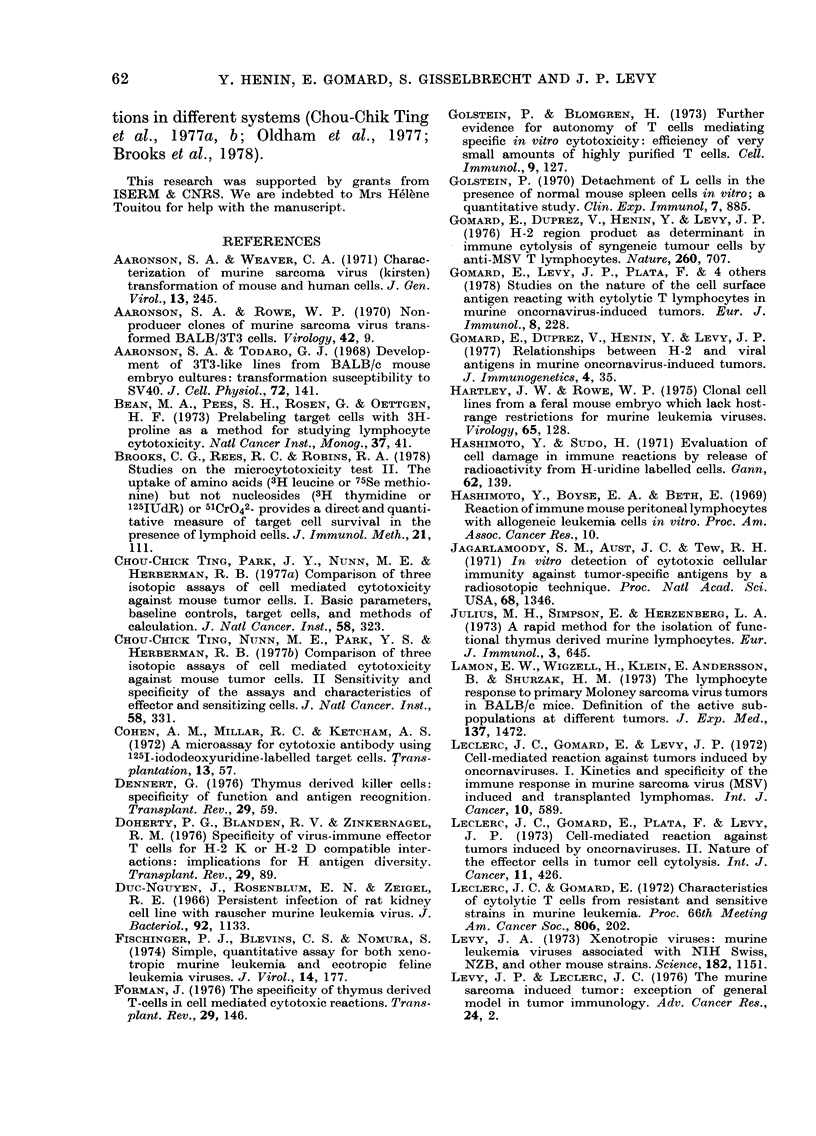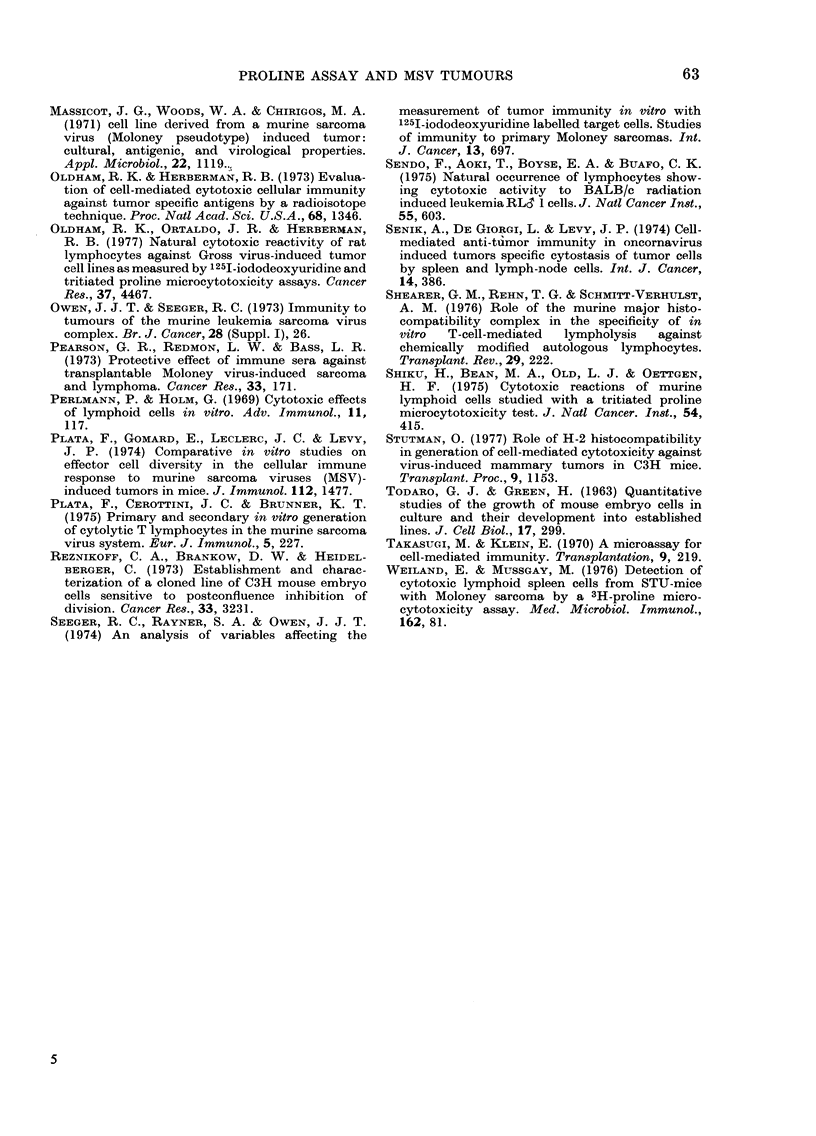# Significance of the proline assay in the study of anti-MSV cell-mediated immune reactions.

**DOI:** 10.1038/bjc.1979.7

**Published:** 1979-01

**Authors:** Y. Henin, E. Gomard, S. Gisselbrecht, J. P. Levy

## Abstract

The cytolysis of 3H-proline-labelled tumour cells growing in monolayer by syngeneic immune lymphocytes has been studied in the murine sarcoma virus (MSV) system. Results show that the proline assay (PA) is a convenient way to reveal the activity of cytolytic T lymphocytes against FMR-like antigens. Using the same effector and target cells, the classical chromium-release test (CRT) fails to reveal any cytolytic activity, and the visual microcytotoxicity assay as well as several derived isotopic methods are known to reveal mainly non-specific reactions due to non-T effector cells. The PA, therefore, appears to be a useful method for testing an antitumour reaction against tumour cells in monolayer. The results are, however, different from those obtained in the CRT using the same effector cells but lymphoma cells in suspension as targets, the major discrepancies being the following: (a) the PA does not provide truly quantitative data, due to the very high lymphoid: effector cell ratios needed in this test; (b) unexpected patterns of antigenic specificities are sometimes detected in PA; (c) a non-specific natural killer activity of non-T cells is frequently detected in the PA, masking at low lymphoid: target cell ratios the T-dependent specific cytolysis; (d) the H-2 restriction of the cytolytic T-cell activity is poorly detected in PA, whereas the role of H-2 antigens is clearly shown by blocking experiments using anti-H-2 antibodies.


*Br. J. Cancer* (1979) **39**, 51

### SIGNIFICANCE OF THE PROLINE ASSAY IN THE STUDY OF ANTI-MSV CELL-MEDIATED IMMUNE REACTIONS

Y. HENIN, E. GOMARD, S. GISSELBRECHT AND J. P. LEVY

*From the Laboratoire d'Immunologie et Virologie des Tumeurs, INSERM U-152, Hôpital Cochin, Bâtiment G. Roussy, Paris*

Received 8 September 1978 Accepted 4 November 1978

**Summary.—The cytolysis of $^{3}$H-proline-labelled tumour cells growing in monolayer by syngeneic immune lymphocytes has been studied in the murine sarcoma virus (MSV) system. Results show that the proline assay (PA) is a convenient way to reveal the activity of cytolytic T lymphocytes against FMR-like antigens. Using the same effector and target cells, the classical chromium-release test (CRT) fails to reveal any cytolytic activity, and the visual microcytotoxicity assay as well as several derived isotopic methods are known to reveal mainly non-specific reactions due to non-T effector cells. The PA, therefore, appears to be a useful method for testing an antitumour reaction against tumour cells in monolayer. The results are, however, different from those obtained in the CRT using the same effector cells but lymphoma cells in suspension as targets, the major discrepancies being the following: (a) the PA does not provide truly quantitative data, due to the very high lymphoid: effector cell ratios needed in this test; (b) unexpected patterns of antigenic specificities are sometimes detected in PA; (c) a non-specific natural killer activity of non-T cells is frequently detected in the PA, masking at low lymphoid : target cell ratios the T-dependent specific cytolysis; (d) the H-2 restriction of the cytolytic T-cell activity is poorly detected in PA, whereas the role of H-2 antigens is clearly shown by blocking experiments using anti-H-2 antibodies.**

ONE of the major problems facing tumour immunologists studying cell-mediated reactions results from the disparity in the results with different *in vitro* cytotoxicity assays. Such disparity could be caused by variations in methodological parameters, which include the nature of the target cells and the length of the incubation period *in vitro* with the effector cells. Both the original visual microcytotoxicity assay (MA) (Takasugi & Klein, 1970) and several derived radioisotopic methods (Jagarlamoody *et al.*, 1971; Cohen *et al.*, 1972; Hashimoto & Sudo, 1971; Perlmann & Holm, 1969; Hashimoto *et al.*, 1969; Bean *et al.*, 1973) require long incubation periods and target cells in monolayers. Most of them reveal simultaneously cytostatic and cytolytic activities due to different effector cell populations, including T and non-T cells as it has been shown, for example, in the murine sarcoma virus (MSV) system (Lamon *et al.*, 1973; Plata *et al.*, 1974; Owen & Seeger, 1973). The cytostatic phenomenon mediated by non-T cells being, at least for the main part, non-specific (Owen & Seeger, 1973; Senik *et al.*, 1974) it is often difficult to assess the specificity of the reactions measured by such methods. More antigen-specific reactions are generally obtained using short-term assays with ascitic lymphoma cells or tumour cells cultivated in suspension as targets. Under these conditions, very clear results can be obtained in the MSV system, regardless of the isotopic marker *e.g.*, $^{51}$Cr (Leclerc *et al.*, 1972), $^{125}$IUdR (Oldham & Herberman, 1973) or $^{3}$H-proline (Shiku *et al.*, 1975; Oldham *et al.*, 1977). These tests appear especially valuable in revealing the activity of cytolytic T lymphocytes

(CTL). They are, however, much less efficient with target cells in monolayers. Relatively few *in vitro*-maintained tumour cells being available in suspension, only a limited number of tumour antigens can, therefore, be studied under optimal conditions. The availability of a specific cytotoxic assay using target cells in monolayers but revealing only cytolytic reactions would, therefore, constitute a major advance in tumour immunology.

The proline assay (PA) was initially proposed (Bean *et al.*, 1973) because: (1) $^{3}$H-proline is retained by the target cells longer than $^{51}$Cr or $^{3}$H-thymidine; (2) it is less toxic than $^{125}$IUdR or [$^{3}$H]-TdR; (3) when released by destroyed target cells the label is not re-utilized, because cold proline is present in large excess in the medium; (4) the results are not noticeably affected by proliferation or cytostasis of tumour cells during the incubation, so that only cytolysis is measured. This method appeared, therefore, as a candidate to replace other tests in the study of target cells in monolayers. It has notably been applied in the study of chemically induced sarcomas (Shiku *et al.*, 1975) and the MSV tumour (Weiland & Mussgay, 1976). Here we report results of experiments using the latter system, aimed at determining the nature of effector cells, the level of H-2 restriction of target-cell cytolysis and the antigenic specificities involved. The results show on the one hand that the PA clearly reveals CTL-mediated reactions but, on the other hand, that the results obtained are not identical with those found using the Cr-release test (CRT). The major discrepancies concern the frequent detection of non-T-cell-mediated cytolysis, the lower precision in the quantification of the reactions, the less clear H-2 restriction of the CTL activity, and the unexpected lack of activity of some effector-cell populations. This suggests that some caution is necessary in the interpretation of antitumour reactions measured using PA, further emphasizing the difficulty in comparing antitumour cell-mediated reactions in different assays.

### MATERIALS AND METHODS

*Mice*.—One to 2-months old C57BL/6 (B6) BALB/B, B10.D2 and BALB/c mice were raised in our own colonies.

*Viruses*.—Tumours were induced *in vivo*, either by MSV-Moloney isolate, (maintained *in vivo* by regular acellular passages in newborn B6) or by the Friend leukaemia virus (FLV) (maintained *in vivo* in adult BALB/c). To infect cultured cells *in vitro*, the same agents or *in vitro*-produced viruses were used. The Moloney leukemia virus (MLV) was harvested from the supernatant of virus-infected non-*Fv*.1-restricted 3T3.FL lines, initially derived from NIH Swiss embryos. The Gross leukaemia virus (GLV) was similarly obtained from an *in vitro*-infected SC1 line derived from wild mice, the Rauscher leukaemia virus (RLV) from a BALB/c 3T3 line *in vitro* transformed by a RLV pseudotype of MSV.

*Cell lines*.—Their main characteristics are summarized in Table I.

*Immune lymphocytes*.—Anti-MSV immune lymphocytes were harvested from the spleens of adult mice inoculated 10–15 days before with 0·2 ml of a $10^{-1}$ dilution of the virus. Anti-FLV immune lymphocytes were obtained similarly from the spleen of adult B6, 10–20 days after an 0·1 ml i.p. inoculation of 1/5 diluted FLV. Spleen-cell pellets were incubated 20 sec in distilled water to eliminate red blood cells and the normal osmolarity was then adjusted by adding hypertonic NaCl. The cells were washed in medium and their concentration adjusted to the test density. Normal spleen cells from the same inbred strain of mice were used as controls.

*Effector-cell purification*.—(1) *T cell elimination*. Non-T cells were purified by eliminating Thy-1-2 cells from the whole-spleen cell suspensions using AKR anti-Thy-1-2 serum and rabbit complement. The preparation and specificity of the anti-Thy-1-2 serum, and the technical conditions used have been described previously (Leclerc *et al.*, 1973).

(2) *T-cell enrichment*. T cells were enriched by passing the whole-spleen cell suspensions through nylon-wool columns (Julius *et al.*, 1973). Columns were first incubated for 1 h at 37°C with 25 ml of medium supplemented with foetal calf serum (FCS). After washing,

TABLE I.—*Target cells*

| Cell lines | Reference | Virus produced | Transforming agent | Origin |
|---|---|---|---|---|
| MSB | Pearson *et al.*, 1973 | M-MSV(MLV) | M-MSV† | C57BL/6 |
| MSC | Massicot *et al.*, 1971 | M-MSV(MLV) | M-MSV | BALB/c |
| B6 MEF | Our laboratory | None | None | C57BL/6 |
| NZB, clone S-2 | Levy, 1973 | Xenotropic | None | NZB |
| SWISS 3T3 | Todaro & Green, 1963 | None | None | Non-inbred Swiss |
| BALB 3T3 | Aaronson & Todaro, 1968 | None | None | BALB/c |
| C3H | Reznikoff *et al.*, 1973 | None | None | C3H/He |
| C3H Moloney | Our laboratory | MLV | None | C3H/He |
| K-BALB | Aaronson & Weaver, 1971 | None | Ki-MSV‡ | BALB/c |
| K-BALB-Moloney | Our laboratory | Ki-MSV(MLV) | Ki-MSV | BALB/c |
| MSV-85 | Aaronson & Rowe, 1970 | None | HT-1-MSV§ | BALB/c |
| SC-1 | Hartley & Rowe, 1975 | None | None | Wild mouse |
| CCC $S^+L^-$ | Fischinger *et al.*, 1974 | Xenotropic RD-114 | None | Cat |
| NRK | Duc-Nguyen *et al.*, 1966 | None | None | Rat |

† MSV Moloney isolate.
‡ MSV Kirsten isolate.
§ HT-1 isolate of the M-MSV.

$2\times10^8$ lymphocytes in 2 ml of FCS-supplemented medium were added, and 30 min later they were passed at a rate of 1 ml/min.

(3) *Macrophage elimination*. Macrophages were removed from unfractionated spleen-cell suspensions by: (a) carbonyl iron and magnet treatment; (b) plastic culture-flask adherence at 37°C for 1 h (Golstein & Blomgren, 1973); (c) successive treatment by both methods.

*The $^3$H-proline assay (PA)*.—PA was performed using a slightly modified version of the original method of Bean *et al.*, (1973). Target-cell monolayers at 80% confluence in T-30 flasks were washed twice with minimum Eagle's medium (MEM) lacking non-essential amino acids (including proline) and incubated overnight in MEM lacking non-essential amino acids plus 15% FCS at 37°C in a 5% $CO_2$ atmosphere, in the presence of 100 μCi $^3$H-proline (L-proline $^3$H-5, sp. act.: 22 Ci/mmol, CEA, Gif sur Yvette, France). The following day the culture was washed twice with complete MEM containing 15% FCS and 2% non-essential amino acids and incubated for 20–30 min at 37°C. The cells were detached with 0·05% trypsin, centrifuged 10 min at 800*g*, suspended in 1 ml MEM containing 10% FCS and 1% non-essential amino acids (test medium), and the cell concentration adjusted to $10^5$ cells/ml. Ten μl containing 1000 target cells were distributed with a microlitre syringe into the wells of microtest II plates prefilled with 0·1 ml of test medium. Effector cells were added ~4 h later in 0·1 ml of test medium, the lymphoid target cell (L/T) ratios varying from 25:1 to 300:1. Six to 8 replicates were used for each L/T ratio. After 24–48 h incubation at 37°C under 5% $CO_2$, the plates were inverted, shaken slightly to remove medium, then submerged ×3 in prewarmed PBS containing 10% FCS, and wiped with blotting paper. The cells remaining alive were harvested by adding 250 μl of 0·05% trypsin to each well, and transferred to scintillation vials. The arithmetic mean of 6–8 wells was calculated in order to estimate percentage relative inhibition of tumour cells after incubation with normal or immune lymphocytes, this inhibition being expressed as follows:

$$\text{inhibition} = 100 \times \frac{\begin{pmatrix}\text{mean ct/min after incubation}\\ \text{with normal lymphocytes}\end{pmatrix} - \begin{pmatrix}\text{mean ct/min after incubation}\\ \text{with immune lymphocytes}\end{pmatrix}}{\begin{matrix}\text{mean ct/min after incubation}\\ \text{with normal lymphocytes}\end{matrix}}$$

The activity of the effector-cell suspension was also expressed in lytic units per $10^6$ effector cells (LU) as previously reported (Plata *et al.*, 1975): one LU represents the number of effector cells necessary to decrease by 50% the radioactivity of 1000 target cells. All statistical analyses were performed using Student's *t* test. The levels of significance were expressed as usual: NS = not significant, $* = 0{\cdot}05 > P > 0{\cdot}01$, $** = 0{\cdot}01 > P > 0\ 001$, $*** = P < 0{\cdot}001$.

*The $^{51}$Cr-release test (CRT)* was performed as previously described (Leclerc *et al.*, 1972)

TABLE II.—*Cytolytic activity of B6 anti-MSV spleen cells in the proline assay* 10–15 *days after virus inoculation*

| | Spleen cell:target cell ratio | | | | | | | | |
|---|---|---|---|---|---|---|---|---|---|
| | 100:1 | | | 200:1 | | | 300:1 | | |
| Target cells | Normal Spleen cells | Immune spleen cells | Inhibition %‡ | Normal spleen cells | Immune spleen cells | Inhibition % | Normal spleen cells | Immune spleen cells | Inhibition % |
| Expt 1: | | | | | | | | | |
| MSB | 4777±462† | 2426±779 | 49·2*** | 4537± 633 | 1880±554 | 59·6*** | 4184±368 | 1154±276 | 72·4*** |
| MEF | NT‖ | NT | | 4621±1011 | 4761±207 | 0 | 4751±263 | 4481±475 | 5·7 NS§ |
| Expt 2: | | | | | | | | | |
| MSB | 3495±512 | 3473±733 | 0 | 3941±693 | 3283±872 | 16·7* | 4572±320 | 2210±365 | 48·1*** |
| MEF | NT | NT | | 739±183 | 811±121 | 0 | 799±199 | 917±269 | 0 |
| Expt 3: | | | | | | | | | |
| MSB | 4930±950 | 5265±672 | 0 | 5357±510 | 4046±628 | 24·5*** | 5842±962 | 2854±407 | 51·1*** |
| MEF | NT | NT | | 9219±997 | 8877±954 | 0 | 8932±903 | 8706±854 | 2·5 NS |

† Arithmetic mean ct/min of 6–8 replicates ±s.d.

‡ Percentage reduction of target-cell radioactivity calculated as described in Materials and Methods. The asterisks indicate the reduction of ct/min significant by Student's *t* test * $=0\cdot05>P>0\cdot01$

** $=0\cdot01>P>0\cdot001$

*** $=P<0\cdot001$

§ Not significant.

‖ Not tested.

using as target cells the B6 Moloney-virus-induced MBL2 lymphoma cells.

*Blocking of the cytolysis by antisera.*—1000 target cells in 10 μl of medium containing 10% FCS and 1% non-essential amino acids were incubated for 2 h at 37°C in 5% $CO_2$ in the presence of 0·1 ml of normal inactivated or immune serum diluted 1: 2 or medium alone. The sera were then removed using a Pasteur pipette, and $3\times10^5$ effector cells in 0·1 ml were added to each well. After 24 h the final activity was calculated by comparing the cytolysis of target cells in the presence of normal or immune serum.

### RESULTS

### *Levels and specificity of the anti-MSV reactions detected in PA*

The spleen cells of MSV regressors, harvested at the beginning of tumour rejection some 12–16 days after virus inoculation, were always effective in PA against MSV tumour cells, when 300: 1 L/T ratios were used (Table II). With decreasing ratios, lower levels of cytolytic activity were detected. When aliquots of the same effector and target-cell preparations were tested in CRT, no activity was found (Table III). Nevertheless, the same effector-cell preparations were always highly efficient in CRT against MBL2 lymphoma cells in suspension. The maximum cytolytic activity detected against MSV tumour cells in monolayer (PA) or against the antigenically related MBL2 cells in suspension (CRT) were in the same range, but when different L/T ratios were used in both tests it appeared that, in terms of LU/$10^6$ effector cells, CRT was more sensitive than PA. However, the latter method was able to show cytolysis of MSV tumour cells, which CRT failed to detect.

TABLE III.—*Cytolytic activity of MSV-infected B6 mouse spleen cells as detected by 3 different assays*

| Spleen cell: target cell ratio | % reduction of target-cell radioactivity: CRT with MBL2 target cells† | CRT with MSB target cells‡ | PA with MSB target cells§ |
|---|---|---|---|
| Expt 1 | | | |
| 200:1 | 45·6‖ | 0‖ | 59·6‖ |
| 100:1 | 43·0 | 0 | 49·2 |
| 50:1 | 22·4 | 0 | 16·1 |
| 20:1 | 13·3 | 0 | 0 |
| Expt 2 | | | |
| 200:1 | 59·8 | 0 | 39·0 |
| 100:1 | 57·5 | 0 | 21·5 |
| 50:1 | 23·2 | 0 | 7·3 |
| 20:1 | 17·2 | 0 | 0 |

† 10,000 per well.

‡ 5000 per well.

(† and ‡:) (a) incubated overnight in test medium. (b) labelled *in situ* by adding 5 μCi of $^{51}Cr$ for 1 h. (c) after washing, effectors cells were added to each well.

§ 1000 MSB per well

‖ Calculated as described in Materials and Methods after 18 h incubation with the 3 assays.

A good level of cytolysis was also found in PA when normal mouse embryonic fibroblasts (MEF) infected *in vitro* with different transforming or non-transforming type C viruses, were used as target cells (Table IV). MLV, FLV or RLV-infected cells were regularly lysed, whereas normal MEF or GLV-infected MEF were unaffected. From these results it can be suggested that: (a) an "FMR-like" antigen could be involved in PA as in classical CRT (Gomard *et al.*, 1978) and (b) an MSV-specific, or tumour-specific antigen is certainly not concerned, since not only MSV tumour cells but also type C virus-infected but non-transformed cells can function as targets in PA.

A somewhat more surprising result was found when anti-MSV and anti-FLV effectors were compared in PA against MSV tumour cells. Whereas anti-FLV and anti-MSV lymphocytes behave similarly in CRT against MBL2 target cells (Table V), B6 anti-FLV were normally inactive in PA, or remained much less efficient than anti-MSV lymphocytes. These results could be taken to suggest that the antigen recognized on MBL2 cells was lacking from the surface of MSV-transformed cells. This hypothesis is, however, unlikely since: (a) the same anti-FLV lymphocytes were also inefficient against FLV-infected mouse embryonic fibroblasts; (b) 6-days *in vitro* coculture of anti-FLV lympho-

TABLE IV.—*Cytolytic activity (as % inhibition‡) of anti-MSV spleen cells detected in PA against MSB or type C virus-infected MEF*

| Spleen cells§ | Target cells† MSB | Normal MEF | MLV-infected MEF | FLV-infected MEF | R-MSV-infected MEF | GLV-infected MEF |
|---|---|---|---|---|---|---|
| Expt 1 | 46·7*** | 0 | 36·6*** | 21·2*** | — | 13·3 NS |
| Expt 2 | 34·6*** | — | — | 57·2*** | 51·0*** | — |
| Expt 3 | 47·2*** | — | 33·1*** | 35·8* | 12·7* | 12·6 NS |
| Expt 4 | 28·6*** | — | — | 42·0*** | — | 17·5 NS |
| Expt 5 | 34·6*** | 0 | — | 57·3*** | 51·1*** | — |

† See Table I.
‡ See footnotes ‡ and § of Table II.
§ Spleen cell: target cell ratio is 300:1 Target cells = $10^4$.

TABLE V.—*Comparison of cytolytic activity of anti-MSV and anti-FLV spleen cells detected in PA and CRT*

| Spleen-cell donors | | Activity in PA on MSB target cell: Effector: target ratio 300:1 ct/min‡ | Inhibition %‡ | Effector: target ratio 200:1 ct/min | Inhibition % | Activity in CRT on MBL2 target cell Effector: target ratio 100:1† $^{51}Cr$ release |
|---|---|---|---|---|---|---|
| Expt 1 | | | | | | |
| Normal | B6 | 4259±860 | | 3941±893 | | |
| Anti-MSV | B6 | 2210±365 | 48·1*** | 3283±872 | 16·7*** | 61·2 |
| Anti-FLV | B6 | 4747±689 | 0 | 3902±827 | 0 | 64·3 |
| Expt 2 | | | | | | |
| Normal | B6 | 4776±480 | | 5447±629 | | |
| Anti-MSV | B6 | 2552±559 | 46·6*** | 4481±987 | 17·7*** | 45·5 |
| Anti-FLV | B6 | 3798±689 | 20·4*** | 5026±510 | 0 | 47·5 |
| Expt 3 | | | | | | |
| Normal | B6 | 5092±704 | | 4912±955 | | |
| Anti-MSV | B6 | 3328±559 | 34·6*** | 2452±494 | 50·1*** | 31·3 |
| Anti-FLV | B6 | 5696±836 | 0 | 4198±869 | 14·5 NS | 31·7 |

† Optimal ratio—Similar activity found at 200:1 or 300:1.

‡ See footnotes †, ‡ and § of Table II.

cytes and MSV tumour cells resulted in a strong secondary cytolytic activity against MBL2 target cells (results not given).

*Nature of the effector cells in PA*

The cytolytic activity being specifically abrogated by anti-Thy-1-2 and complement treatment of the effector cells, it appeared dependent on the presence of T lymphocytes (Table VI). Macrophages were apparently not concerned, since carbonyl iron and magnet treatments did not significantly decrease attacker-cell efficiency. The role of a non-phagocytic but plastic-adherent cell cannot be ruled out, since the activity of the whole-spleen cell suspension was clearly decreased by plastic adherence (Table VI) and still more by plastic adherence plus carbonyl iron treatment. It must be noted, however, that after such treatments the activity of the treated cells was always greater if measured in a 48-h rather than in a 24-h assay. This suggested that relatively time-consuming and aggressive manipulations could have non-specifically altered the effector cells, which then need more than 24 h to restore their normal functions. This hypothesis was reinforced by the fact that passing through nylon-wool

TABLE VI.—*Effect of different treatments on the activity of anti-MSV spleen cells tested in PA against MSB target cells*

| Spleen cells treated with† | Incubation (h) | ct/min with normal lymphocytes‡ | ct/min with immune lymphocytes‡ | Inhibition‡ % |
|---|---|---|---|---|
| Expt 1 | | | | |
| Normal AKR Serum+C′ | 24 | 11451±1981 | 7935±1413 | 30·7*** |
| Anti-Thy1-2 Serum+C′ | 24 | 10571±1375 | 10685±1818 | 0 |
| Normal AKR Serum+C′ | 48 | 5197± 581 | 2351± 463 | 54·7*** |
| Anti-Thy1-2 Serum+C′ | 48 | 5883± 388 | 5868±1083 | 0 |
| Expt 2 | | | | |
| Test medium | 24 | 1331± 175 | 620± 62 | 53·4*** |
| Carbonyl iron | 24 | 1617± 200 | 789± 212 | 51·2*** |
| Adherence on plastic | 24 | 1683± 110 | 1343± 110 | 20·2*** |
| Carbonyl iron+adherence on plastic | 24 | 1428± 460 | 1371± 206 | 3·9 NS |
| Test medium | 48 | 1097± 126 | 375± 49 | 65·2*** |
| Carbonyl iron | 48 | 1025± 161 | 550± 94 | 46·3*** |
| Adherence on plastic | 48 | 1271± 178 | 993± 203 | 21·9*** |
| Carbonyl iron+adherence on plastic | 48 | 1143± 38 | 991± 150 | 13·3* |
| Expt 3 | | | | |
| Test medium | 24 | 1115± 183 | 407± 99 | 63·5*** |
| Passage through nylon-wool column | 24 | 1392± 168 | 617± 116 | 55·7*** |
| Test medium | 48 | 440± 34 | 255± 73 | 42·0*** |
| Passage through nylon-wool column | 48 | 494± 125 | 290± 108 | 41·3*** |

† See Material and Methods.

‡ See footnotes †, ‡ and § of Table II.

columns, which takes only a relatively short time, did not decrease the cytolytic activity of the spleen-cell suspensions. We concluded, therefore, that the CTL were, at least for the most part, the effector cells of the anti-MSV reaction measured in PA.

The role of cytolytic T lymphocytes (CTL) would be further supported by the existence of an H-2 restriction of cytolytic activity, this property being one of the major characteristics of CTL in the MSV (Gomard *et al.*, 1976) as well as in many other systems (Doherty *et al.*, 1976; Dennert 1976; Forman 1976; Shearer *et al.*, 1976). The experiments reported in Table VII showed that such an H-2 restriction can be found in PA, allogeneic MSV tumour cells being lysed significantly but at a 2–3-times lower level than syngeneic targets. However, we have never found in PA, the very strong H-2 restriction which is regularly detected in CRT with lymphoma target cells (Gomard *et al.*, 1978).

The involvement of H-2 normal antigens in the effector–target-cell interaction is clearly confirmed by the observation that preincubation of H-$2^b$ tumour cells with anti-H-$2^b$ antibodies specifically abrogated their sensitivity to syngeneic anti-MSV effector cells in PA (Table VIII) as previously shown in the CRT (Gomard *et al.*, 1977).

*Detection of natural killer (NK) cells in PA*

The cytolytic activities of non-immune spleen cells were measured in PA by comparing cytolysis in the presence of normal lymphoid cells and in medium alone. The results in Table IX show that in PA normal murine spleen cells had a strong killer activity for: (a) normal mouse fibroblasts; (b) type C virus-infected murine cells whether transformed or untransformed; (c) normal xenogeneic cells. This NK-cell activity was not H-2 restricted, and did not depend on a viral antigen, since it was also found with non-virus-infected cells. It was not dependent on tumour antigen(s) since normal cells were also affected. It appeared, therefore, as mainly non-specific and predominantly determined by the general sensitivity of the target cells to immune cytolysis, a

TABLE VII.—*Cytolysis of MSV-transformed cells by syngeneic or allogeneic effector cells*

| | | | Target cells | | | | | |
|---|---|---|---|---|---|---|---|---|
| | | | MSB | | | MSC | | |
| Spleen-cell donors | H-2 Haplotype | Effector: target ratio | ct/min | Inhibition† % | Lytic‡ units | ct/min | Inhibition† % | Lytic‡ units |
| Normal mice | *b*/*d*§ | 300 | 4184±368 | | | 759±156 | | |
| | | 200 | 4537±633 | | | 840±112 | | |
| | | 100 | 4777±422 | | | 930± 79 | | |
| | | 50 | 4502±425 | | | 1156± 40 | | |
| B6 anti-MSV | *b* | 300 | 1154±276 | 72·4*** | | 300± 28 | 60·4*** | |
| | | 200 | 1830±554 | 59·6*** | 9·9 | 373± 44 | 55·5*** | 5·8 |
| | | 100 | 2426±779 | 49·2*** | | 630±106 | 32·2*** | |
| | | 50 | 3775±497 | 16·1*** | | 857± 54 | 25·8*** | |
| BALB/c anti-MSV | *d* | 300 | 2619±519 | 37·4*** | | 409± 81 | 46·1*** | |
| | | 200 | 3042±125 | 32·9*** | 1·6 | 471± 72 | 43·9*** | 2·5 |
| | | 100 | 4389±874 | 8·2 NS | | 686±146 | 26·2*** | |
| | | 50 | 4101±239 | 8·8 NS | | 877± 53 | 24·1*** | |
| B10.D2 anti-MSV | *d* | 300 | 1368±447 | 67·3*** | | 306± 47 | 59·6*** | |
| | | 200 | 1697±175 | 62·5*** | 7·4 | 364± 59 | 56·6*** | 16·6 |
| | | 100 | 2800±411 | 41·3*** | | 401± 38 | 56·8*** | |
| | | 50 | 3605±720 | 19·9* | | 599±129 | 48·1*** | |
| BALB/B anti-MSV | *b* | 300 | 2523±393 | 36·9*** | | 547± 69 | 27·9* | |
| | | 200 | 3255±319 | 28·2*** | 2·3 | 646±208 | 23·1* | 0 |
| | | 100 | 3531±628 | 26·1* | | 769+139 | 17·9* | |
| | | 50 | 4197±328 | 6·7 NS | | 1066±149 | 7·8 NS | |

† See footnotes †, ‡ and § of Table II.
‡ See Material and Methods.
§ *b* with MSB targets and *d* with MSC targets.

TABLE VIII.—*Effect of anti-H-2$^b$ serum on the activity in PA of H-2$^b$ anti-MSV spleen cells*

| | Spleen cell: target cell ratio | | | | | |
|---|---|---|---|---|---|---|
| | 300:1 | | | 200:1 | | |
| MSB Target cells treated with† | ct/min with normal‡ lymphocytes | ct/min with immune‡ lymphocytes | inhibition‡ % | ct/min with normal lymphocytes | ct/min with immune lymphocytes | inhibition % |
| Expt 1 | | | | | | |
| Test medium | 6723±782 | 5483±822 | 18·4*** | | | |
| Normal mouse serum | 4282±597 | 3373±595 | 21·2*** | | | |
| BALB/c anti-BALB/B serum | 3801±654 | 3545±361 | 6·7 NS | | | |
| Expt 2 | | | | | | |
| Normal mouse serum | 2167±360 | 784±372 | 63·8*** | 2250±293 | 1432±324 | 36·4*** |
| BALB/c anti-BALB/B serum | 1428±382 | 1357±400 | 4·9 NS | 1842±474 | 1810±344 | 1·7 NS |

† See Material and Methods.

‡ See footnotes †, ‡ and § of Table II.

clear parallelism existing, for example, between the sensitivity to NK cells and the sensitivity to related anti-H-2 lymphocytes. Table X shows that the effector cells were non-phagocytic and non-T. It is important to emphasize that aliquots of the same effector-cell populations were always devoid of cytolytic activities when tested in CRT against ascitic tumour cells (results not given).

### DISCUSSION

The above-reported results show that PA is a convenient method of revealing

TABLE IX.—*Spontaneous cytolytic activity of normal spleen cells in PA (Inhibition %)*‡

Target cells†

(a) Normal mouse cell lines

| Spleen-cell donors | NZB (H-2$^{d}$) | Swiss (H-2$^{s}$) | BALB 3T3 (H-2$^{d}$) | C3H (H-2$^{k}$) | SC-1 (H-2?) |
|---|---|---|---|---|---|
| Normal BALB/c | 0 | 69·3*** | 43·0*** | 0 | 61·2*** |
| Normal CBA | 0 | 68·5*** | 78·1*** | 10·8 NS | NT |
| Normal C3H/He | 0 | 50·0*** | 60·4*** | 8·0 NS | 75·0*** |
| Normal DBA/2 | 0 | 74·9*** | 73·8*** | 11·5 NS | 24·9*** |
| Normal Swiss | 0 | 74·2*** | 53·4*** | 0 | 29·1*** |
| Normal B6 | NT‖ | NT | NT | NT | 29·1*** |
| Normal AKR | 0 | 70·4*** | 64·8*** | 10 NS | NT |
| B6 anti-BALB/c¶ | 0 | 0 | 75·3*** | 0 | NT |
| B6 anti-AKR¶ | 0 | 0 | NT | 0 | NT |

(b) transformed and/or type C virus-producer cell lines

| Spleen-cell donors | K-BALB/c (H-2$^{d}$) | MLV-infected K-BALB/c (H-2$^{d}$) | MSB (H-2$^{b}$) | MLV infected C3H-MSV (H-2$^{k}$) | MSV-85 (H-2$^{d}$) |
|---|---|---|---|---|---|
| Normal B6 | 37·3*** | 24·2*** | NT | 17·0*** | 19·0*** |
| Normal BALB/c | 46·3*** | 40·9*** | 0 | 16·6*** | 23·9*** |
| Normal AKR | NT | NT | 29·7*** | NT | NT |
| Normal CBA | NT | NT | 48·4*** | NT | NT |
| Normal C3H/He | NT | NT | 28·2*** | NT | NT |
| Normal DBA/2 | NT | NT | 45·8*** | NT | NT |
| Normal Swiss | NT | NT | 23·1*** | NT | NT |
| BALB/c anti B6¶ | NT | NT | 78·4*** | NT | NT |
| B6 anti BALB/c¶ | NT | NT | NT | NT | 23·9*** |

(c) Xenogeneic cell lines

| Spleen-cell donors | SIRC (Rabbit) | NRK (Rat) | CCC S$^{+}$L$^{-}$ (Cat) |
|---|---|---|---|
| Normal BALB/c | 0 | 48·5*** | 83·3*** |
| Normal C3H/He | 0 | 46·7*** | 83·2*** |
| Normal DBA/2 | 0 | 36·5*** | 56·5*** |
| Normal Swiss | 0 | 33·8*** | 83·1*** |
| Normal B6 | 0 | 33·0*** | 81·1*** |

† See Table I.

‡ Inhibition percentage was calculated as follows:

$$100 \times \frac{\text{mean ct/min after incubation with test medium-mean ct/min after incubation with normal lymphocytes}}{\text{mean ct/min after incubation with test medium}}$$

‖ NT = not tested.

¶ 50 × 10$^{6}$ normal spleen cells inoculated i.p. 4 days before.

specific cell-mediated cytolytic reactions against MSV tumour cells. The cytolysis induced by immune lymphocytes is predominantly, if not exclusively, due to T cells. The possible involvement of a minor population of plastic-adherent cells does not change this conclusion, since its activity was abrogated by an anti-Thy 1-2 and complement treatment, suggesting that it too may be a T-cell subpopulation. The cytolytic activity appears very specific and probably directed against the same "FMR-like" antigen which is recognized by anti-MSV CTL in CRT (Gomard *et al.*, 1978).

Despite the use of tumour target cells in monolayers and of relatively long *in vitro* incubations, PA gives much more specific results than MA. The fact that PA does not measure cytostatic phenomena is probably determinant (Seeger *et al.*, 1974). Moreover, according to the recent results of Brooks *et al.* (1978) we may suppose that the use of labelled amino acids in place of radioactive nucleotides explains its better specificity than that of other isotopic MA. PA allows one to detect the cytolysis of MSV tumour cells themselves, whereas CRT fails to do so, or provides only very weak and hardly reproducible results. This advantage of PA contrasts with its lower sensitivity than classical

TABLE X.—*Effect of different treatments of normal spleen cells on their activity in PA against MSB and target cells*

| Spleen cells treated with† | MSB incubation (h) | MSB ct/min test medium | MSB ct/min with normal lymphocytes | MSB inhibition‡ % | MSC incubation (h) | MSC ct/min with test medium | MSC ct/min with normal lymphocytes | MSC inhibition % |
|---|---|---|---|---|---|---|---|---|
| Expt 1: | | | | | | | | |
| no spleen cell | 24 | 5552±1023 | | | 24 | 2406±254 | | |
| test medium | 24 | | 3642±609 | 34·4*** | 24 | | 1331±175 | 44·7*** |
| carbonyl iron | 24 | | 3482±437 | 37·3*** | 24 | | 1617±200 | 32·8*** |
| carbonyl iron+adherence on plastic | 24 | | | | | | 1428±460 | 40·6*** |
| adherence on plastic | 24 | | 3586±913 | 35·4 | 24 | | 1683±224 | 30·0*** |
| no spleen cell | | | | | 48 | 1962±236 | | |
| test medium | | | | | 48 | | 1077±126 | 45·1*** |
| carbonyl iron | | | | | 48 | | 1025±161 | 47·1*** |
| carbonyl iron+adherence on plastic | | | | | 48 | | 1143± 38 | 41·7*** |
| adherence on plastic | | | | | 48 | | 1271±178 | 35·2*** |
| Expt 2: | | | | | | | | |
| no spleen cell | 24 | 7292±1918 | | | | | | |
| normal serum | 24 | | 5725±990 | 21·5* | | | | |
| anti-Thy 1·2 serum+C′ | 24 | | 5285±687 | 21·5* | | | | |
| no spleen cell | 48 | 1620±316 | | | 48 | 3157±354 | | |
| test medium | 48 | | 1359±323 | 16·1* | 48 | | 1941±286 | 38·5*** |
| normal serum | 48 | | 1306±308 | 19·4* | | | | |
| normal serum+C′ | 48 | | 1271±165 | 21·5* | 48 | | 1639±509 | 48·0*** |
| anti-Thy 1·2 serum+C′ | 48 | | 1309±163 | 19·2* | 48 | | 1904±297 | 36·7*** |
| Expt 3: | | | | | | | | |
| no spleen cell | 24 | 2273±253 | | | | | | |
| test medium | 24 | | 1930±311 | 13·4* | | | | |
| passage through nylon-wool column | 24 | | 1954±375 | 14·0* | | | | |
| no spleen cell | 48 | 888 ±154 | | | | | | |
| test medium | 48 | 569±131 | 569±131 | 35·0*** | | | | |
| passage through nylon-wool column | 48 | | 604±137 | 32·0*** | | | | |

† See Material and Methods.

‡ See footnote ‡ of Table IX.

CRT using lymphoma cells as target. It is probably related to the longer *in vitro* incubations which are possible with PA, allowing lysis of relatively insensitive tumour cells. Such incubations are hardly possible in CRT due to the high level of spontaneous marker elution, except when specially selected tumour cells are used. Moreover, the cellular lesion which is necessary to allow the detachment of altered cells from the plastic could be an earlier step in cell death than the release of $^{51}$Cr-labelled large molecules. PA appears, therefore, as a useful test in tumour immunology when tumour-cell populations growing in monolayer have to be used.

Nevertheless, it must be emphasized that in the MSV system, PA and CRT, which both reveal a CTL-mediated reaction directed against an "FMR-like" antigen, do not provide identical results. At least 3 major differences have been detected in our experiments:

(1) A natural killer activity was regularly found in PA but not in CRT. It has been detected, however, by others with the latter method (Bean *et al.*, 1973; Oldham *et al.*, 1977). The discrepancy is probably related to the variable sensitivity of tumour cells to NK-cell-mediated cytolysis, with an especially high sensitivity of *in vitro* tumour cells (Sendo *et al.*, 1975). The fact that PA measures the cells unsticking could account for its specially high ability to reveal natural killing since it is well established that non-T-cells are frequently responsible for such phenomena, independent of target-cell cytolysis (Golstein, 1970). The important point is that the NK-cell-mediated reactions could mask, in PA experiments much more than in CRT, weak CTL-mediated cytolysis.

(2) Anti FLV-effector cells were much less efficient in PA than anti-MSV, whereas both kinds of CTL behave alike in CRT. The reasons for this surprising phenomenon is unclear, and the role of different cell-surface antigens appears unlikely, as discussed above. An explanation may perhaps be found in the high degree of adhesiveness of anti-FLV-CTL. It is known that these cells can be retained by nylon-wool columns (Leclerc & Gomard, 1972) and we observed that they were much more adherent to target-cell monolayers than anti-MSV-CTL. Whatever its origin, this phenomenon could alter the CTL-tumour-cell interaction and it could be responsible for false-negative reactions suggesting an incorrect pattern of antigen specificity.

(3) The H-2 restriction of CTL reactivity was far weaker in PA than in CRT. This could be related to quantitation problems, since when strongly efficient CTL are tested the H-2 restriction can only be established by quantitative experiments using several L/T ratios (Gomard *et al.*, 1978). Such experiments are hardly possible in PA since the cytotoxic activity falls very rapidly with decreasing L/T ratios. The NK-cell activity is also increased by prolonged *in vitro* incubations. This NK activity, which increases the background cytolysis, can in turn mask some of the specific cytolysis, especially at low L/T ratios when syngeneic but not allogeneic effector cells should be efficient at a relatively weak level. PA, therefore, appears *a priori* to be a bad method for revealing H-2 restriction phenomena, and this may explain the weakness of the H-2 restriction of viral mammary-tumour cell cytolysis previously reported (Stutman, 1977). It is remarkable, in view of the above considerations, that it was nevertheless possible to establish that H-2 antigens were involved in PA reactivity, as in the CRT, as demonstrated by the strong blocking activity of anti-H-2 antibodies.

In conclusion, PA can be a useful method of testing CTL-mediated anti-tumour or anti-viral immune reactions, but several peculiarities of the test should be borne in mind in order to avoid misinterpretation of the results. Once again, it appears that the technical parameters are especially determinant in the detection of cellular anti-tumour reactions, as recently emphasized by other investigations in different systems (Chou-Chik Ting *et al.*, 1977*a*, *b*; Oldham *et al.*, 1977; Brooks *et al.*, 1978).

This research was supported by grants from ISERM & CNRS. We are indebted to Mrs Hélène Touitou for help with the manuscript.

### REFERENCES

AARONSON, S. A. & WEAVER, C. A. (1971) Characterization of murine sarcoma virus (kirsten) transformation of mouse and human cells. *J. Gen. Virol.*, **13**, 245.

AARONSON, S. A. & ROWE, W. P. (1970) Non-producer clones of murine sarcoma virus transformed BALB/3T3 cells. *Virology*, **42**, 9.

AARONSON, S. A. & TODARO, G. J. (1968) Development of 3T3-like lines from BALB/c mouse embryo cultures: transformation susceptibility to SV40. *J. Cell. Physiol.*, **72**, 141.

BEAN, M. A., PEES, S. H., ROSEN, G. & OETTGEN, H. F. (1973) Prelabeling target cells with 3H-proline as a method for studying lymphocyte cytotoxicity. *Natl Cancer Inst., Monog.*, **37**, 41.

BROOKS, C. G., REES, R. C. & ROBINS, R. A. (1978) Studies on the microcytotoxicity test II. The uptake of amino acids ($^{3}$H leucine or $^{75}$Se methionine) but not nucleosides ($^{3}$H thymidine or $^{125}$IUdR) or $^{51}CrO_4{}^{2-}$ provides a direct and quantitative measure of target cell survival in the presence of lymphoid cells. *J. Immunol. Meth.*, **21**, 111.

CHOU-CHICK TING, PARK, J. Y., NUNN, M. E. & HERBERMAN, R. B. (1977*a*) Comparison of three isotopic assays of cell mediated cytotoxicity against mouse tumor cells. I. Basic parameters, baseline controls, target cells, and methods of calculation. *J. Natl Cancer. Inst.*, **58**, 323.

CHOU-CHICK TING, NUNN, M. E., PARK, Y. S. & HERBERMAN, R. B. (1977*b*) Comparison of three isotopic assays of cell mediated cytotoxicity against mouse tumor cells. II Sensitivity and specificity of the assays and characteristics of effector and sensitizing cells. *J. Natl Cancer. Inst.*, **58**, 331.

COHEN, A. M., MILLAR, R. C. & KETCHAM, A. S. (1972) A microassay for cytotoxic antibody using $^{125}$I-iododeoxyuridine-labelled target cells. *Transplantation*, **13**, 57.

DENNERT, G. (1976) Thymus derived killer cells: specificity of function and antigen recognition. *Transplant. Rev.*, **29**, 59.

DOHERTY, P. G., BLANDEN, R. V. & ZINKERNAGEL, R. M. (1976) Specificity of virus-immune effector T cells for H-2 K or H-2 D compatible interactions: implications for H antigen diversity. *Transplant. Rev.*, **29**, 89.

DUC-NGUYEN, J., ROSENBLUM, E. N. & ZEIGEL, R. E. (1966) Persistent infection of rat kidney cell line with rauscher murine leukemia virus. *J. Bacteriol.*, **92**, 1133.

FISCHINGER, P. J., BLEVINS, C. S. & NOMURA, S. (1974) Simple, quantitative assay for both xenotropic murine leukemia and ecotropic feline leukemia viruses. *J. Virol.*, **14**, 177.

FORMAN, J. (1976) The specificity of thymus derived T-cells in cell mediated cytotoxic reactions. *Transplant. Rev.*, **29**, 146.

GOLSTEIN, P. & BLOMGREN, H. (1973) Further evidence for autonomy of T cells mediating specific *in vitro* cytotoxicity: efficiency of very small amounts of highly purified T cells. *Cell. Immunol.*, **9**, 127.

GOLSTEIN, P. (1970) Detachment of L cells in the presence of normal mouse spleen cells *in vitro*; a quantitative study. *Clin. Exp. Immunol*, **7**, 885.

GOMARD, E., DUPREZ, V., HENIN, Y. & LEVY, J. P. (1976) H-2 region product as determinant in immune cytolysis of syngeneic tumour cells by anti-MSV T lymphocytes. *Nature*, **260**, 707.

GOMARD, E., LEVY, J. P., PLATA, F. & 4 others (1978) Studies on the nature of the cell surface antigen reacting with cytolytic T lymphocytes in murine oncornavirus-induced tumors. *Eur. J. Immunol.*, **8**, 228.

GOMARD, E., DUPREZ, V., HENIN, Y. & LEVY, J. P. (1977) Relationships between H-2 and viral antigens in murine oncornavirus-induced tumors. *J. Immunogenetics*, **4**, 35.

HARTLEY, J. W. & ROWE, W. P. (1975) Clonal cell lines from a feral mouse embryo which lack host-range restrictions for murine leukemia viruses. *Virology*, **65**, 128.

HASHIMOTO, Y. & SUDO, H. (1971) Evaluation of cell damage in immune reactions by release of radioactivity from H-uridine labelled cells. *Gann*, **62**, 139.

HASHIMOTO, Y., BOYSE, E. A. & BETH, E. (1969) Reaction of immune mouse peritoneal lymphocytes with allogeneic leukemia cells *in vitro*. *Proc. Am. Assoc. Cancer Res.*, 10.

JAGARLAMOODY, S. M., AUST, J. C. & TEW, R. H. (1971) *In vitro* detection of cytotoxic cellular immunity against tumor-specific antigens by a radiosotopic technique. *Proc. Natl Acad. Sci.* USA, **68**, 1346.

JULIUS, M. H., SIMPSON, E. & HERZENBERG, L. A. (1973) A rapid method for the isolation of functional thymus derived murine lymphocytes. *Eur. J. Immunol.*, **3**, 645.

LAMON, E. W., WIGZELL, H., KLEIN, E. ANDERSSON, B. & SHURZAK, H. M. (1973) The lymphocyte response to primary Moloney sarcoma virus tumors in BALB/c mice. Definition of the active subpopulations at different tumors. *J. Exp. Med.*, **137**, 1472.

LECLERC, J. C., GOMARD, E. & LEVY, J. P. (1972) Cell-mediated reaction against tumors induced by oncornaviruses. I. Kinetics and specificity of the immune response in murine sarcoma virus (MSV) induced and transplanted lymphomas. *Int. J. Cancer*, **10**, 589.

LECLERC, J. C., GOMARD, E., PLATA, F. & LEVY, J. P. (1973) Cell-mediated reaction against tumors induced by oncornaviruses. II. Nature of the effector cells in tumor cell cytolysis. *Int. J. Cancer*, **11**, 426.

LECLERC, J. C. & GOMARD, E. (1972) Characteristics of cytolytic T cells from resistant and sensitive strains in murine leukemia. *Proc. 66th Meeting Am. Cancer Soc.*, **806**, 202.

LEVY, J. A. (1973) Xenotropic viruses: murine leukemia viruses associated with NIH Swiss, NZB, and other mouse strains. *Science*, **182**, 1151.

LEVY, J. P. & LECLERC, J. C. (1976) The murine sarcoma induced tumor: exception of general model in tumor immunology. *Adv. Cancer Res.*, **24**, 2.

MASSICOT, J. G., WOODS, W. A. & CHIRIGOS, M. A. (1971) cell line derived from a murine sarcoma virus (Moloney pseudotype) induced tumor: cultural, antigenic, and virological properties. *Appl. Microbiol.*, **22**, 1119.

OLDHAM, R. K. & HERBERMAN, R. B. (1973) Evaluation of cell-mediated cytotoxic cellular immunity against tumor specific antigens by a radioisotope technique. *Proc. Natl Acad. Sci. U.S.A.*, **68**, 1346.

OLDHAM, R. K., ORTALDO, J. R. & HERBERMAN, R. B. (1977) Natural cytotoxic reactivity of rat lymphocytes against Gross virus-induced tumor cell lines as measured by $^{125}$I-iododeoxyuridine and tritiated proline microcytotoxicity assays. *Cancer Res.*, **37**, 4467.

OWEN, J. J. T. & SEEGER, R. C. (1973) Immunity to tumours of the murine leukemia sarcoma virus complex. *Br. J. Cancer*, **28** (Suppl. I), 26.

PEARSON, G. R., REDMON, L. W. & BASS, L. R. (1973) Protective effect of immune sera against transplantable Moloney virus-induced sarcoma and lymphoma. *Cancer Res.*, **33**, 171.

PERLMANN, P. & HOLM, G. (1969) Cytotoxic effects of lymphoid cells *in vitro*. *Adv. Immunol.*, **11**, 117.

PLATA, F., GOMARD, E., LECLERC, J. C. & LEVY, J. P. (1974) Comparative *in vitro* studies on effector cell diversity in the cellular immune response to murine sarcoma viruses (MSV)-induced tumors in mice. *J. Immunol.* **112**, 1477.

PLATA, F., CEROTTINI, J. C. & BRUNNER, K. T. (1975) Primary and secondary *in vitro* generation of cytolytic T lymphocytes in the murine sarcoma virus system. *Eur. J. Immunol.*, **5**, 227.

REZNIKOFF, C. A., BRANKOW, D. W. & HEIDELBERGER, C. (1973) Establishment and characterization of a cloned line of C3H mouse embryo cells sensitive to postconfluence inhibition of division. *Cancer Res.*, **33**, 3231.

SEEGER, R. C., RAYNER, S. A. & OWEN, J. J. T. (1974) An analysis of variables affecting the measurement of tumor immunity *in vitro* with $^{125}$I-iododeoxyuridine labelled target cells. Studies of immunity to primary Moloney sarcomas. *Int. J. Cancer*, **13**, 697.

SENDO, F., AOKI, T., BOYSE, E. A. & BUAFO, C. K. (1975) Natural occurrence of lymphocytes showing cytotoxic activity to BALB/c radiation induced leukemia RL♂ 1 cells. *J. Natl Cancer Inst.*, **55**, 603.

SENIK, A., DE GIORGI, L. & LEVY, J. P. (1974) Cell-mediated anti-tumor immunity in oncornavirus induced tumors specific cytostasis of tumor cells by spleen and lymph-node cells. *Int. J. Cancer*, **14**, 386.

SHEARER, G. M., REHN, T. G. & SCHMITT-VERHULST, A. M. (1976) Role of the murine major histocompatibility complex in the specificity of *in vitro* T-cell-mediated lympholysis against chemically modified autologous lymphocytes. *Transplant. Rev.*, **29**, 222.

SHIKU, H., BEAN, M. A., OLD, L. J. & OETTGEN, H. F. (1975) Cytotoxic reactions of murine lymphoid cells studied with a tritiated proline microcytotoxicity test. *J. Natl Cancer. Inst.*, **54**, 415.

STUTMAN, O. (1977) Role of H-2 histocompatibility in generation of cell-mediated cytotoxicity against virus-induced mammary tumors in C3H mice. *Transplant. Proc.*, **9**, 1153.

TODARO, G. J. & GREEN, H. (1963) Quantitative studies of the growth of mouse embryo cells in culture and their development into established lines. *J. Cell Biol.*, **17**, 299.

TAKASUGI, M. & KLEIN, E. (1970) A microassay for cell-mediated immunity. *Transplantation*, **9**, 219.

WEILAND, E. & MUSSGAY, M. (1976) Detection of cytotoxic lymphoid spleen cells from STU-mice with Moloney sarcoma by a $^{3}$H-proline microcytotoxicity assay. *Med. Microbiol. Immunol.*, **162**, 81.